# Functional Characterization of the Steroid Reductase Genes *GmDET2a* and *GmDET2b* from *Glycine max*

**DOI:** 10.3390/ijms19030726

**Published:** 2018-03-03

**Authors:** Weige Huo, Bodi Li, Jiebing Kuang, Pingan He, Zhihao Xu, Jinxiang Wang

**Affiliations:** 1 State Key Laboratory for Conservation and Utilization of Subtropical Agro-bioresources, South China Agricultural University, Guangzhou 510642, China; wghuo@cau.edu.cn (W.H.); 011068ch@stu.scau.edu.cn (B.L.); binel168@163.com (J.K.); pinganhe@stu.scau.edu.cn (P.H.); xuzhihao@stu.scau.edu.cn (Z.X.); 2 Root Biology Center, College of Natural Resources and Environment, South China Agricultural University, Guangzhou 510642, China

**Keywords:** GmDET2, steroid reductase, brassinosteroid, soybean

## Abstract

Brassinosteroids are important phytohormones for plant growth and development. In soybean (*Glycine max*), BR receptors have been identified, but the genes encoding BR biosynthesis-related enzymes remain poorly understood. Here, we found that the soybean genome encodes eight steroid reductases (GmDET2a to GmDET2h). Phylogenetic analysis grouped 105 steroid reductases from moss, fern and higher plants into five subgroups and indicated that the steroid reductase family has experienced purifying selection. GmDET2a and GmDET2b, homologs of the *Arabidopsis* thaliana steroid 5α-reductase AtDET2, are proteins of 263 amino acids. Ectopic expression of *GmDET2a* and *GmDET2b* rescued the defects of the *Atdet2-1* mutant in both darkness and light. Compared to the mutant, the hypocotyl length and plant height of the transgenic lines *GmDET2a* and *GmDET2b* increased significantly, in both darkness and light, and the transcript levels of the BR biosynthesis-related genes *CPD*, *DWF4*, *BR6ox-1* and *BR6ox-2* were downregulated in *GmDET2aOX-23* and *GmDET2bOX-16* lines compared to that in *Atdet2-1*. Quantitative real-time PCR revealed that *GmDET2a* and *GmDET2b* are ubiquitously expressed in all tested soybean organs, including roots, leaves and hypocotyls. Moreover, epibrassinosteroid negatively regulated *GmDET2a* and *GmDET2b* expression. Sulfate deficiency downregulated *GmDET2a* in leaves and *GmDET2b* in leaves and roots; by contrast, phosphate deficiency upregulated *GmDET2b* in roots and leaves. Taken together, our results revealed that GmDET2a and GmDET2b function as steroid reductases.

## 1. Introduction

Brassinosteroids (BRs), as classical phytohormones, play important roles in many aspects of plant growth and development including root growth and development [1,2], stomatal development [3], seed germination [4], skotomorphogenesis [5,6], phototropism [7], nodulation [8], immunity responses [9] and abiotic stress responses [10,11].

In animals, steroid hormones are perceived through nuclear receptors. By contrast, in plants, BR is sensed by the BR receptors such as brassinosteroid insensitive 1 (BRI1) in the plasma membranes [12,13]. Recently, the linear BR signaling pathway in *Arabidopsis* has been revealed. In the absence of BRs, BRI1 is bound by the membrane-located kinase BKI1 [14]. Upon reception of BR, BRI1 disassociates BKI1 [14] and physically interacts with the membrane kinase BAK1, which is considered as a co-receptor [15]. BRI1 and BAK1 transphosphorylate each other [14]. Brassinosteroid insensitive 2 (BIN2) functions downstream of BRI1. As a highly conserved GSK kinase and negative regulator of BR signaling, BIN2 phosphorylates transcription factors brassinazole-resistant 1 (BZR1) and BZR2 and in turn inactivated them [16,17,18,19]. Conversely, protein phosphatase 2A (PP2A) mediates the dephosphorylation of BZR1 to activate it [20]. When BR levels are high, BR signaling leads to the inactivation of BIN2. Accordingly, the dephosphorylated BZR1 and BZR2 shuttle between the cytoplasm and nucleus, binding promoters of several downstream genes, further strengthening BR signaling [21]. BIN2 and BZR1 have been reported to be regulated by the proteasome-dependent pathway in *Arabidopsis* [18,22].

Previous studies have revealed that *Arabidopsis* has three functional BR receptors, namely BRI1, BRL1 and BRL3, while AtBRL2 appears to be nonfunctional in BR signaling [23,24]. The rice genome encodes four BR receptors, OsBRI1, OsBRL1, OsBRL2 and OsBRL3 [25,26]. BR receptors have also been identified in tomato [27], pea [28], barley [29], cotton [30] and soybean [31,32].

As reported, more than 50 BRs have been isolated and identified from several plant species [33]. Among them, brassinolide (BL) is the most active BR. Based on previous studies, the first reaction of BL biosynthesis is the formation of campestanol (CN) from campesterol (CR); the second step is the conversion of CN to castasterone (CS) via C6 oxidation; and the final step is the conversion of CS to BL [34]. The steroid 5α-reductase, DET2, which hydrogenates a (24R)-24-methylcholest-4-en-3-one intermediate to convert CR into CN, catalyzes the rate-limiting step in BR biosynthesis [35,36]. The *Atdet2-1* mutant has a dwarf phenotype; its growth and development in the dark and light both retarded significantly [5].

In 1990, a Japanese group reported that BR promotes adventitious rooting in soybean hypocotyl cuttings [37]. Later, BR was found to promote stem growth in soybean [38], and the application of BR increased soybean tolerance to drought and increased the concentration of soluble sugar and proline [39]. Additionally, BR increases the expression of soybean *small auxin up RNA 6B* (*SAU 6B*) in elongating epicotyls, in a time-dependent manner [38]. These studies indicate that BRs regulate soybean growth, development and stress responses at physiological and molecular levels, but the underlying mechanisms are poorly understood.

Although the BR receptors in soybean have been identified [31,32], BR biosynthesis remains elusive at the molecular level. In this study, we identified eight steroid reductase genes (*GmDET2a* to *GmDET2h*) in soybean. Phylogenetic analysis indicated that a total of 105 steroid reductase (dehydrogenases) from moss, fern and higher plants can be grouped into five subgroups, and steroid reductase is subjected to purifying selection during evolution. *GmDET2a* and *GmDET2b* were negatively regulated by epibrassinosteroid. Phosphate (Pi) starvation induced the expression of *GmDET2b* in leaves and roots, and sulfate deficiency downregulated the abundance of *GmDET2b* in leaves and roots. Ectopic expression of *GmDET2a* and *GmDET2b* rescued the defects of *det2-1* mutants both in darkness and light and altered the transcript levels of BR biosynthesis-related genes.

## 2. Results

### 2.1. Identification of Steroid Reductases in Soybean

To identify steroid reductases in the soybean proteome, we took two approaches. First, we searched the soybean proteome for the PF02544 domain (http://pfam.xfam.org/), the signature domain of steroid reductase [40], and found a total of six putative steroid reductase. Second, the amino acid sequence of the known steroid reductase AtDET2 was used to search the soybean proteome with BLAST-P (http://blast.ncbi.nlm.nih.gov), and the two proteins encoded by *Glyma.07g144400* and *Glyma.11g010000* showed the highest identity and similarity to AtDET2. Collectively, the soybean genome encodes eight steroid reductase (Table 1). Although the PF02544 domain was not found in Glyma.07g144400 and Glyma11g010000, the BLAST search against the soybean proteome with *Arabidopsis* DET2 showed that Glyma.07g144400 and Glyma.11g010000 appear to have a similar function to that of AtDET2, and thus, they have been named GmDET2a and GmDET2b, respectively.

In order of BLAST E-value, six proteins, in addition to Glyma.07g144400 and Glyma.11g010000, were found. In order, they are Glyma.02G081200, Glyma.02G081100, Glyma.11G110300, Glyma.14G043500, Glyma.02g273300 and Glyma.19g261600. The six related genes have been named *GmDET2c*, *GmDET2d*, *GmDET2e*, *GmDET2f*, *GmDET2g* and *GmDET2h*, respectively, as listed in Table 1. *GmDET2c*, *GmDET2d* and *GmDET2g* are located on chromosome 2, *GmDET2b* and *GmDET2e* on chromosome 11, *GmDET2a* on chromosome 7, *GmDET2f* on chromosome 12 and *GmDET2h* on chromosome 19. The amino acid length of steroid reductases in soybean ranged between 263 and 342 amino acids.

**Figure 1:**
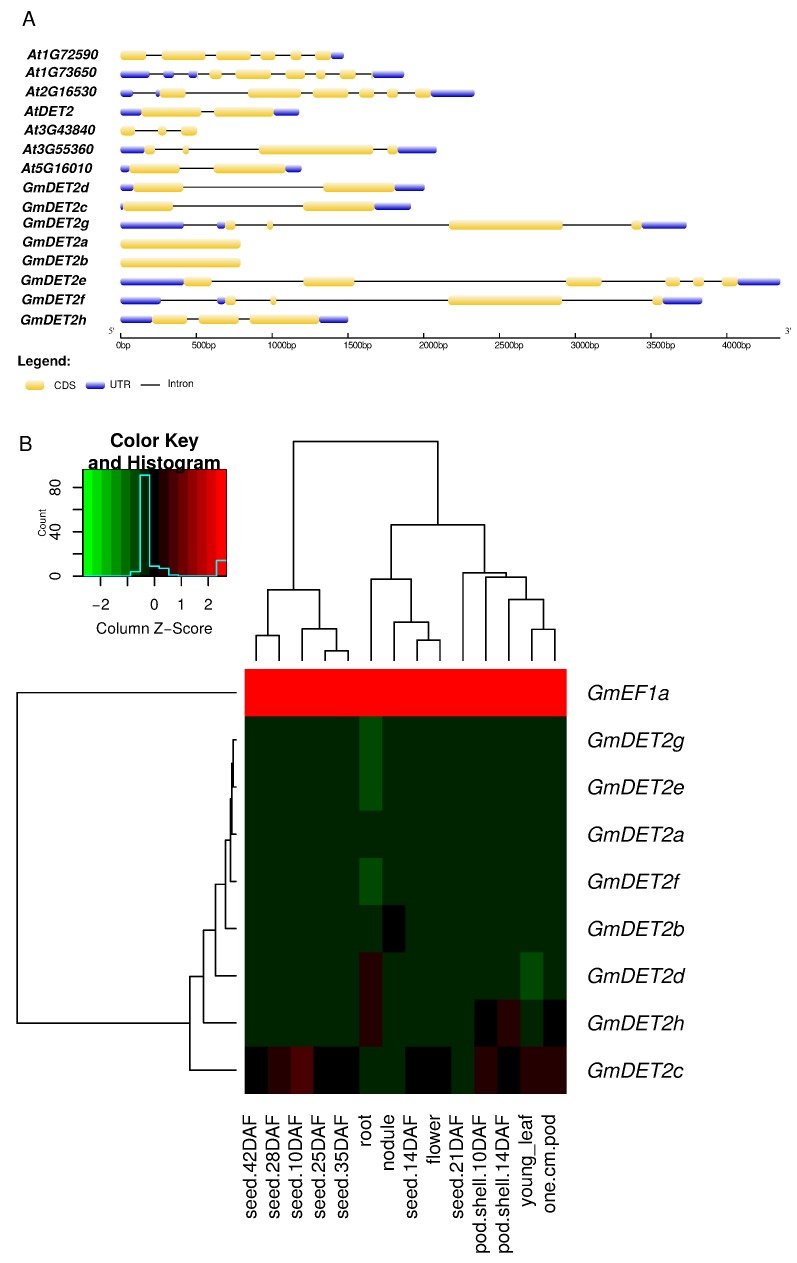
Gene structure of *Arabidopsis* and soybean steroid reductase and transcript levels of the soybean steroid reductase family. The gene structure of seven and eight steroid reductase from *Arabidopsis* and soybean are shown (**A**). CSDS2.0 was used to analyze the CDS (coding DNA sequence), UTR (untranslated region) and intron of these genes. The heat map (**B**) demonstrates the expression levels of *GmDET2a* to *GmDET2h*. The normalized expression data from RNA-Seq were downloaded from SoyBase [41]. DAF, day after fertilization.

Based on the data in Phytozome (www.phytozome.org), the gene structure of soybean DETs was analyzed with the GSDS2.0 program (http://60.209.29.212/). The soybean *DET2* family can be grouped into three subgroups (intro-less, one intron and two or more introns); both *GmDET2a* and *GmDET2b* are intron-less genes; *GmDET2c* and *GmDET2d* have one intron; while *GmDET2e*, *GmDET2f*, *GmDET2g* and *GmDET2h* contain 3, 5, 4 and 2 introns, respectively (Figure 1A).

Based on previous RNA-Seq study on soybean [41], we found that *GmDET2a* and *GmDET2b* are ubiquitously expressed in young leaves, flowers, pods, seeds, roots and nodules, with relatively higher transcript abundance in roots and nodules (Figure 1). The transcript levels of *GmDET2d* and *GmDET2h* are higher in roots than in other organs, while those of *GmDET2b* and *GmDET2d* are higher in nodules. The transcripts of *GmDET2c* and *GmDET2h* are higher in leaves, and the expression levels of *GmDET2c* and *GmDET2h* are higher in flowers. Interestingly the transcripts of all eight steroid reductase genes can be detected in seeds, with transcripts of *GmDET2c* in 10 days after fertilization (DAF) seeds being significantly higher. The abundance of *GmDET2c* and *GmDET2h* is somewhat higher in one-cm pod, pod shell 10 DAF and pod shell 14 DAF (Figure 1B).

### 2.2. Phylogenetic Analysis of Steroid Reductases

**Figure 2:**
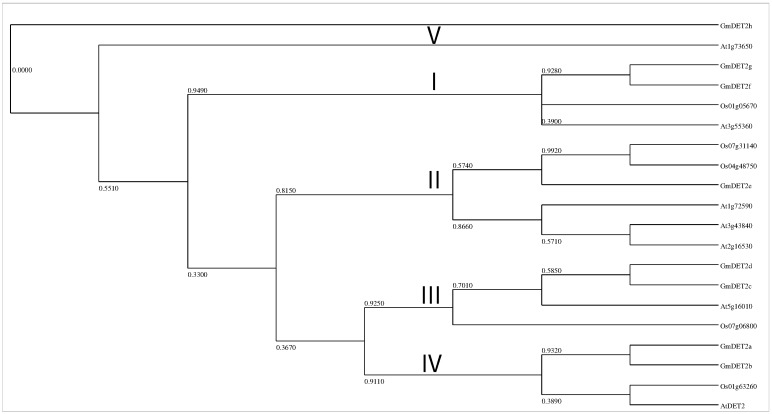
Molecular Phylogenetic analysis by the maximum likelihood method. The evolutionary history was inferred by using the maximum likelihood method based on Jones et al.’s w/freq. model [44]. The tree with the highest log likelihood (−2073.6839) is shown. The percentage of trees in which the associated taxa clustered together is shown next to the branches. Initial tree(s) for the heuristic search were obtained automatically by applying the neighbor-join and BioNJalgorithms to a matrix of pairwise distances estimated using a JTTmodel and then selecting the topology with the superior log likelihood value. A discrete gamma distribution was used to model evolutionary rate differences among sites (five categories (+G, parameter = 5.1876)). The tree is drawn to scale, with branch lengths measured in the number of substitutions per site. The analysis involved 20 amino acid sequences. All positions containing gaps and missing data were eliminated. There was a total of 74 positions in the final dataset. Evolutionary analyses were conducted in MEGA6 [42]. Steroid reductases from soybean, *Arabidopsis* and rice can be grouped into five subgroups (I, II, III, IV and V).

We aligned the amino acid sequences of eight soybean DET2s with 12 homologs from the reference species *Arabidopsis* thaliana and *Oryza sativa* and reconstructed the phylogenetic tree of DET2s with MEGA6 [42]. As expected, GmDET2a and GmDET2b grouped together with AtDET2 and Os01g63260. GmDET2c and GmDET2d clustered with At5g16010, GmDET2e with Os04g48750, GmDET2f and GmDET2g with At3G55360 and GmDET2h with At1g73650. In short, DET2s from soybean, *Arabidopsis* and rice can be grouped into five sub-clades (Figure 2).

**Figure 3:**
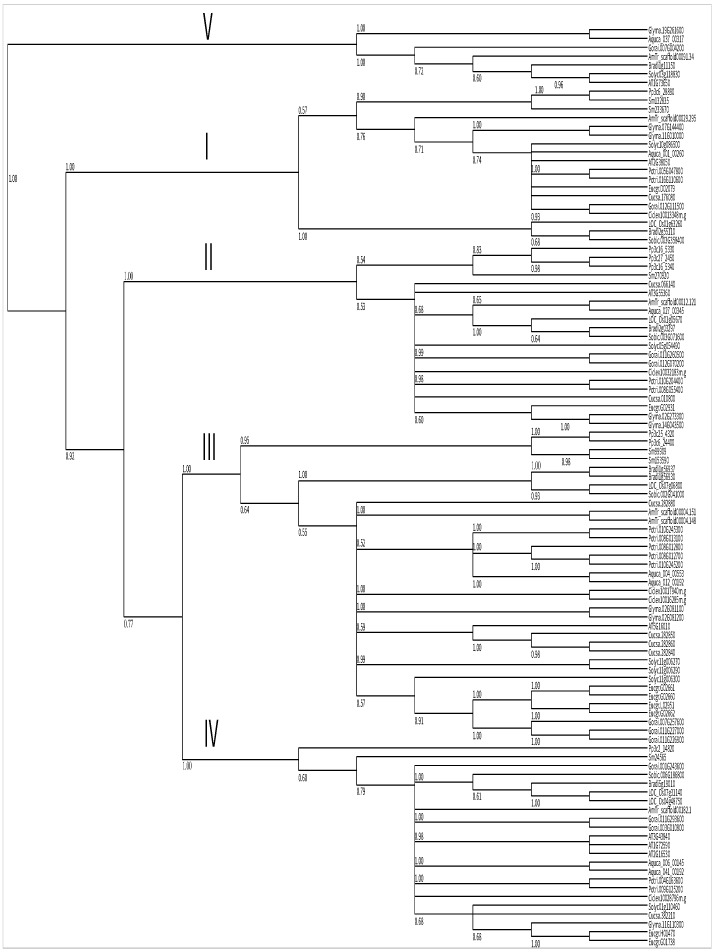
Molecular phylogenetic analysis of steroid reductases in planta. BLASTP was used to search for BR receptor homologues in different plant species. A total of 105 steroid reductase as shown in Additional File 1 (Spreadsheet S1) was analyzed from *Physcomitrella patens* (Pp), *Selaginella moellendorffii* (Sm), *Cucumis sativus* (Cucsa), *Populus trichocarpa* (Potri), *Eucalyptus grandis* (Eucgr), *Citrus clementina* (Cicle), *Solanum lycopersicum* (Solyc), *Glycine max* (Glyma), *Arabidopsis thaliana* (AT), *Brachypodium distachyon* (Bradi), *Oryza sativa* (Os), *Amborella trichopoda* (AmTr), *Gossypium raimondii* (Gorai), *Aquilegia coerulea* (Aqcoe) and *Sorghum bicolor* (Sobic). Values expressed below the branches are the probability of the bootstrap value with 1000 repeats. MrBayes 3.2 [43] software was used to reconstruct the phylogenetic tree, as described in the Methods Section.

To extensively decipher the evolutionary history of steroid reductase in the kingdom Plantae, we further collected steroid reductases from multiple plants, including *Physcomitrella patens*, *Selaginella moellendorffii*, *Oryza sativa*, *Brachypodium distachyon*, *Sorghum bicolor*, etc. After alignment with the CLUSTALW program, sequences were analyzed with MrBayes 3.2 [43]. As shown in Figure 3, steroid reductases of Plantae are grouped into five subgroups. Of note, except for group V , all subgroups have members from *Physcomitrella patens* and *Selaginella moellendorffii*. This indicates that higher plants have retained ancestral steroid reductase genes and that steroid reductase plays an important role in growth and development.

**Figure 4:**
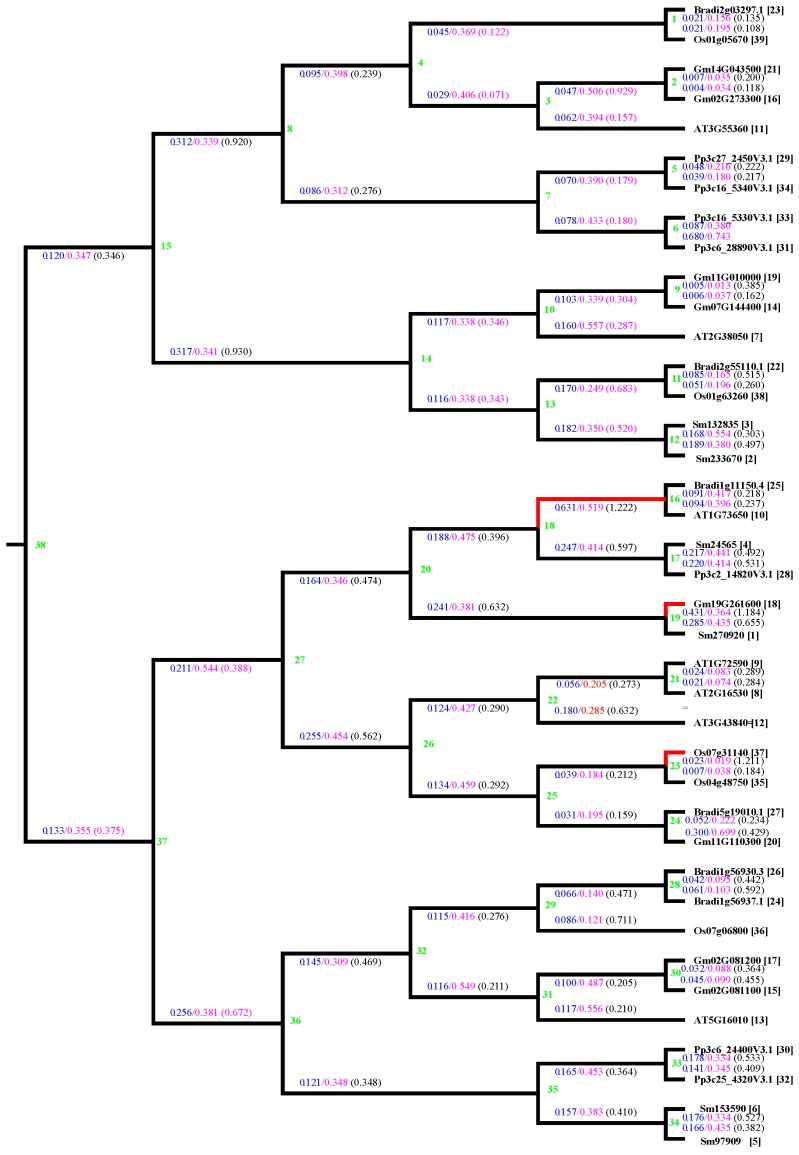
Estimation of Ka/Ks in steroid reductase genes from soybean, common bean, *Arabidopsis*, rice. The cDNA sequences and amino acid sequences of steroid reductase from moss *Physcomitrella patens* (Pp), fern *Selaginella moellendorffii* (Sm), *Glycine max* (Gm), *Arabidopsis thaliana* (AT), *Brachypodium distachyon* (Bradi) and *Oryza sativa* (Os) were used to estimate Ka/Ks (http://services.cbu.uib.no/tools/kaks). The Ka and Ks values in each node and branch are marked in blue and red, respectively. A total of 38 nodes is shown in green, and the ratios of Ka/Ks in each branch are indicated in parentheses. The ratios of Ka/Ks in most branches are less than one, except for three branches in red.

To identify the selection pressure during evolution of plant steroid reductases, we explored the ratios of Ka to Ks (Ka/Ks) of steroid reductase from *P. patens*, *S. moellendorffii*, *O. sativa*, *B. distachyon*, *G. max* and *A. thaliana*. Generally, genes are subjected to three kinds of selection, namely positive selection (Ka/Ks > 1), neutral selection (Ka/Ks = 1), and negative or purifying selection (Ka/Ks < 1). Results in Figure 4 showed that plant steroid reductase are subjected to purifying selection, except for three branches marked in red, in which the Ka/Ks are greater than one (Figure 4).

### 2.3. Transcript Levels of *GmDET2a* and *GmDET2b* in the Seedling Stage

**Figure 5:**
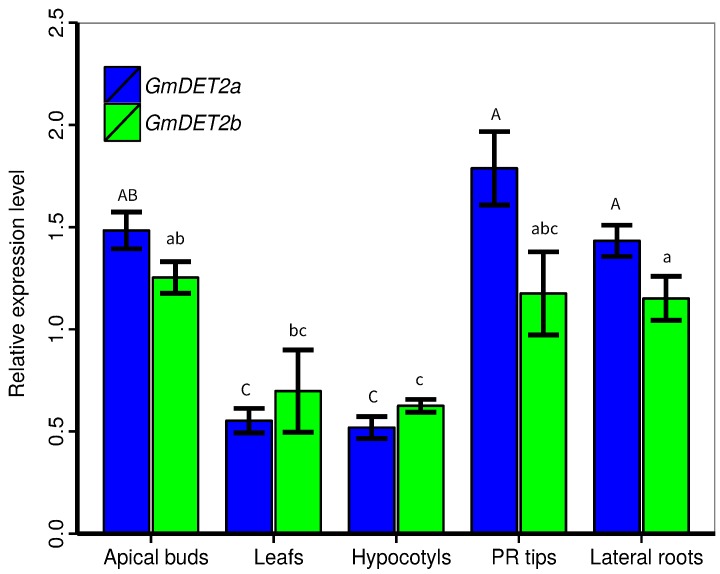
Transcript levels of *GmDET2a* and *GmDET2b* in soybean seedlings. RNA was extracted from seven-day-old soybean YC03-3. Quantitative real-time PCR was exploited to detect the transcripts of *GmDET2a* (in blue) and *GmDET2b* (in green) in apical buds, leaves, hypocotyls, primary root (PR) tips and lateral roots. Results are the means ± SD from four replicates. One-way ANOVA was used to compare the differences between organs, with uppercase letters or lowercase letters indicating the difference at the 0.05 level.

Taking into consideration the important roles of brassinosteroid in plant growth and development, that *GmDET2* appear to be involved in BR biosynthesis, and that *GmDET2a* and *GmDET2b* are the closest homologs of *AtDET2*, we thus focused on them in subsequent experiments. We first employed quantitative real-time PCR (qRT-PCR) to detect the transcripts of *GmDET2a* and *GmDET2b* in the seedlings of soybean. As shown in Figure 5, qRT-PCR results showed that *GmDET2a* and *GmDET2b* were both expressed in apical buds, leaves, hypocotyls, primary root (PR) tips and lateral roots. The transcripts of *GmDET2a* in apical buds, PR tips and lateral roots are somewhat higher than those in leaves and hypocotyls (Figure 5). A similar pattern was observed in the case of *GmDET2b* (Figure 5).

### 2.4. *GmDET2a* and *GmDET2b* Were Negatively Regulated by Epibrassinosteroid

**Figure 6:**
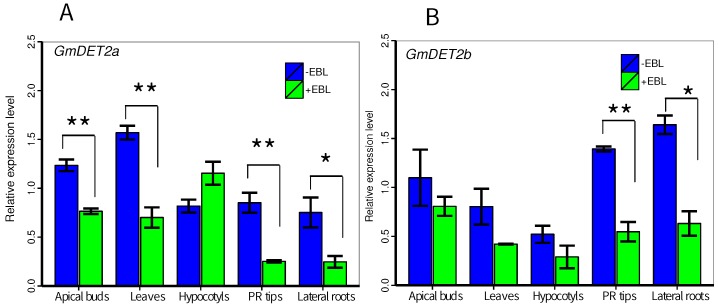
Responses of *GmDET2a* and *GmDET2b* to exogenous epibrassinosteroid (EBL) in soybean seedlings. RNA was extracted from five-day-old soybean YC03-3 treated with 10 μM EBL for 6 h or not. Quantitative real-time PCR was exploited to detect the transcripts of *GmDET2a* (**A**) and *GmDET2b* (**B**) in apical buds, leaves, hypocotyls, primary root (PR) tips and lateral roots. Results are the means ± SD from 4 replicates. Student’s *t*-test was used to compare the differences between EBL treatment and control, respectively (*, 0.01<p<0.05; **, 0.001<p<0.01).

As previously reported, *AtDET2* is negatively regulated by exogenous BR [45,46]. To explore this relationship in soybean, we looked at the expression pattern of *GmDET2a* and *GmDET2b* in soybean seedlings treated with epibrassinosteroid (EBL) for 6 h. As shown in Figure 6A, in general, *GmDET2a* was negatively regulated by 6 h EBL treatment. The abundance of *GmDET2a* was significantly reduced in apical buds (p<0.01), leaves (p<0.01), primary root (PR) tips (p<0.01) and lateral roots (p<0.05), but not negatively regulated by EBL in hypocotyls. *GmDET2b* was negatively regulated by EBL in only primary root tips (p<0.01) and lateral roots (p<0.05), but not in apical buds, leaves and hypocotyls (Figure 6B).

### 2.5. Responses of *GmDET2a* and *GmDET2b* to Nutrient Deficiencies

**Figure 7:**
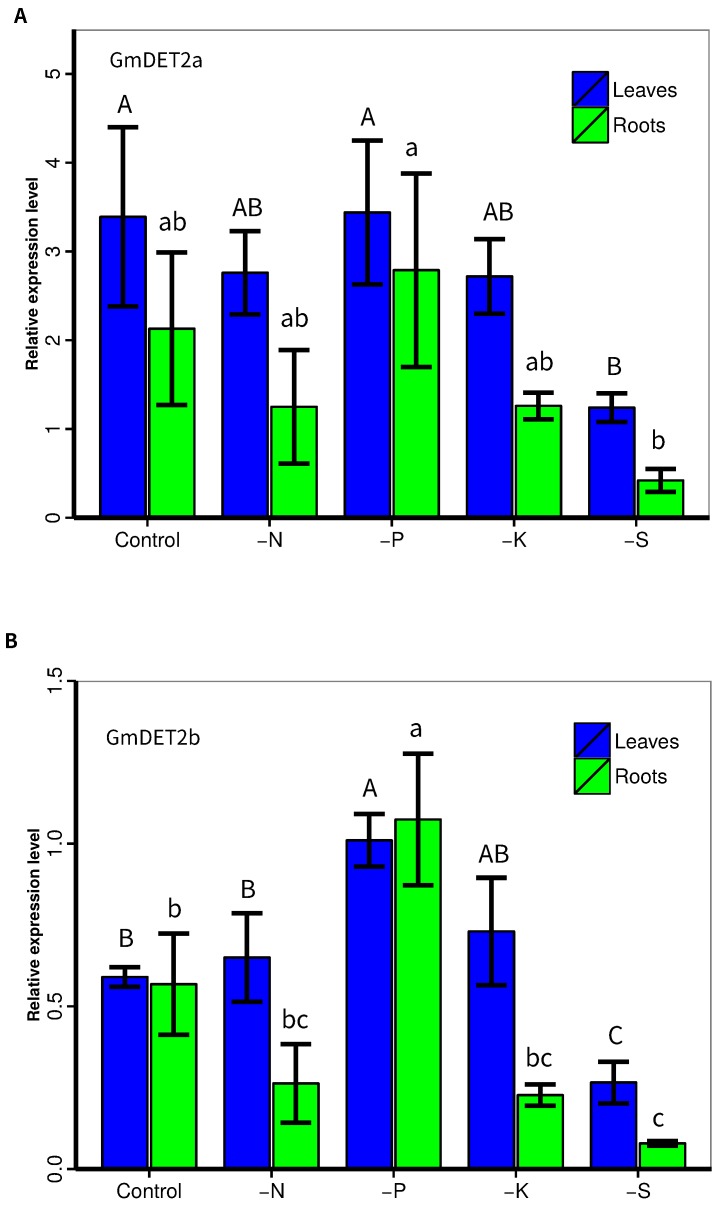
Responses of *GmDET2a* and *DET2b* to nutrient deficiency. Soybean YC03-3 was hydroponically cultured. After seven days, when the seedlings developed the first true leaf, soybean plants were transferred into a control nutrient solution, Pi-depleted (-P), nitrogen-depleted (-N), potassium-depleted (-K) and sulfate-depleted (-S) solution for 14 days. Quantitative real-time PCR was used to detect the expression levels of *GmDET2a* (**A**) and *GmDET2b* (**B**); *GmEF1a* was used as a reference gene. Results are the means ± SD from four replicates. One-way ANOVA was employed to compare the difference, with uppercase letters or lowercase letters indicating the difference at the 0.05 level. L, leaves; R, roots.

As our group is interested in plant nutrition, we also explored the responses of *GmDET2a* (Figure 7A) and *GmDET2b* (Figure 7B) to nutrient deficiencies. Soybean seedlings were starved of phosphate (-P), nitrogen (-N), potassium (-K) or sulfate (-S) for 14 days. Analysis by qRT-PCR showed that the transcripts of *GmDET2a* in leaves were decreased by -S, and those of *GmDET2b* in leaves and roots were reduced by -S (p<0.05; Figure 7B), while being upregulated by -P in both leaves and roots (p<0.05). The transcripts of *GmDET2a* and *GmDET2b* were not regulated by -N or -K in either leaves or roots.

### 2.6. Ectopic Expression of *GmDET2a* and *GmDET2b* in *Atdet2-1* Rescued the Growth and Development Defects

A loss of function mutation in *DET2* affects the growth and development of *Arabidopsis* in both darkness and light [5,35]. To determine the functions of *GmDET2a* and *GmDET2b*, we constructed over-expression plasmids with the pCHF3 vector and transformed the loss-of-function mutant, *Atdet2-1* [5]. We selected transgenic lines and identified single-copy lines based on the segregation ratio of 3:1. The transcript levels of *GmDET2a* and *GmDET2b* were explored in transgenic lines *GmDET2aOX-20*, *GmDET2aOX-23*, *GmDET2bOX-16* and *GmDET2bOX-23*. As shown in Figure S1, the transgenic lines of *GmDET2a* and *GmDET2b* were overexpressing lines.

**Figure 8:**
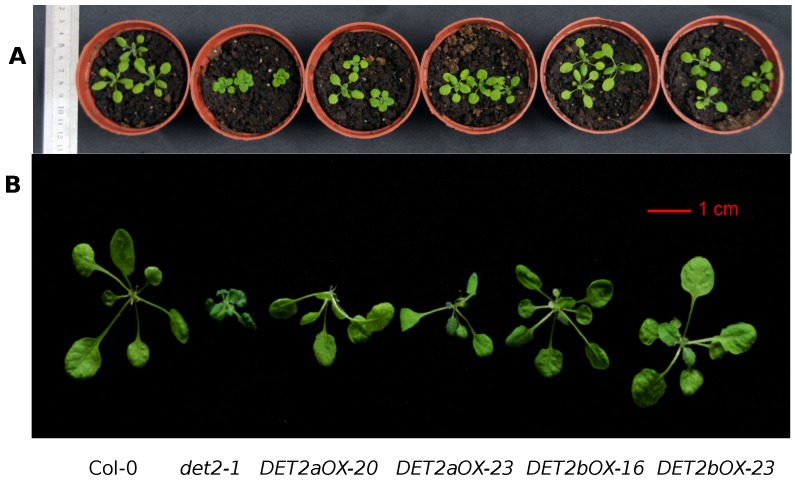
*GmDET2a* and *GmDET2b* rescued the leaf defects of *Atdet2-1*. Ectopic expression of *GmDET2a* or *GmDET2b* rescued the leaf defects in *Atdet2-1* background. *Arabidopsis* seedlings were cultured in soils for 15 days (**A**) or 30 days (**B**), respectively. Scale bar, 1 cm.

Consistent with previous results [5], the leaves of *Atdet2-1* mutant are curled with a shorter petiole, as compared to wild-type (WT, Columbia (Col-0)) (Figure 8). As expected, when overexpressing *GmDET2a* or *GmDET2b* in *Atdet2-1*, the defective leaf growth and development were rescued. Images were taken after 15 days (Figure 8A) and 30 days of germination (Figure 8B).

**Figure 9:**
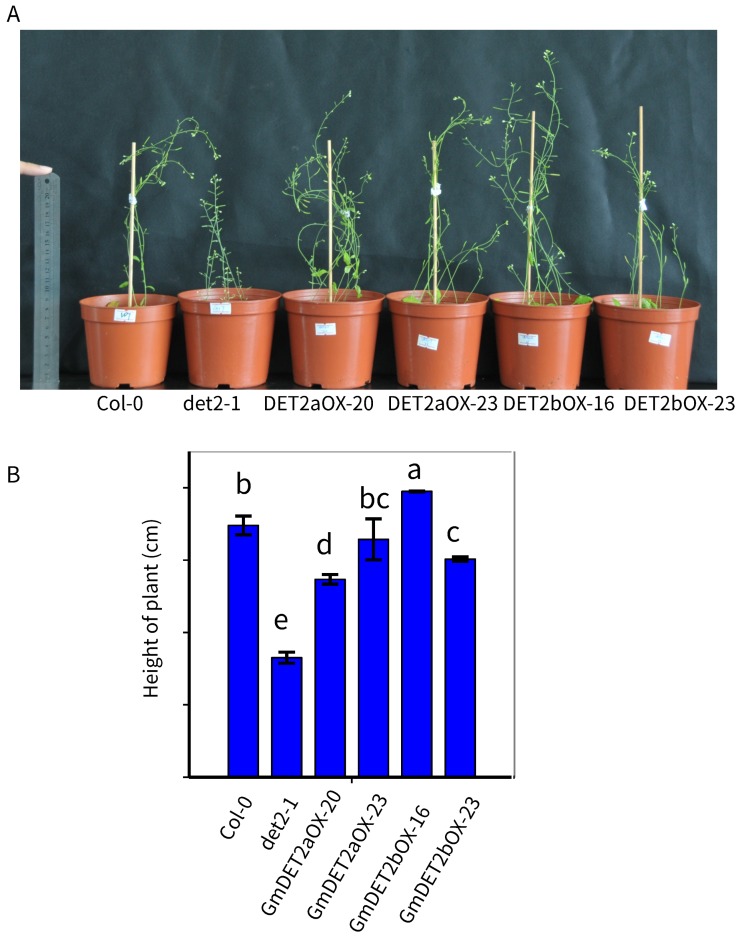
Ectopic expression of *GmDET2a* and *GmDET2b* rescued the height of *Atdet2-1*. The height of 50-day-old wild-type Col-0, *Atdet2-1* and transgenic lines were measured (**A**). Results are the means ± SD from four independent experiments, and one-way ANOVA was used to detect the differences among Col-0, *Atdet2-1* and transgenic lines in the *det2-1* background (**B**), with different letters indicating significant differences at the 0.05 level.

Consistent with previous study, the *Atdet2-1* mutant is dwarf with short siliques (Figure 9A). We measured the height of transgenic *Arabidopsis* after 50 days of germination. The height of transgenic lines is higher in contrast to *Atdet2-1* (p<0.05, Figure 9B). This indicates that like AtDET2, GmDET2a or GmDET2B complement AtDET2, a steroid reductase.

**Figure 10:**
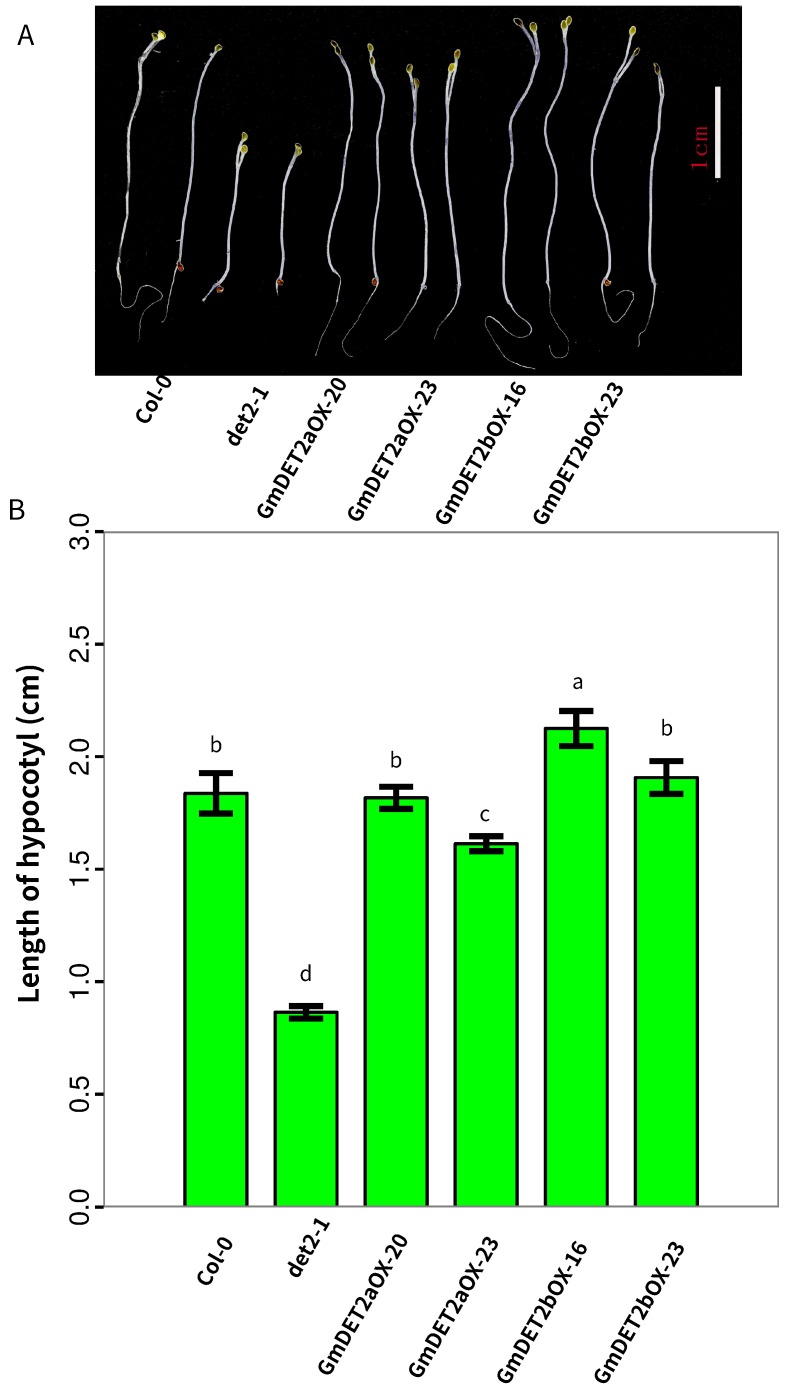
Ectopic expression of *GmDET2a* and *GmDET2b* rescued the skotomorphogenesis of *Atdet2-1*. Seeds of Col-0, *Atdet2-1*, transgenic lines were sown in half strength MS media and cultured in a growth chamber for one day with 16 h light/8 h dark cycle to promote germination. Seeds were then cultured in the dark for five days and photographed (**A**). Scale bar, 1 cm. The length of hypocotyls (**B**) was determined with ImageJ. Results are the means ± SD from four replicates with 10 samples, respectively. Different letters indicate significant differences at the 0.05 level (one-way ANOVA).

BRs have been documented to play crucial roles in skotomorphogenesis [12], and the *Atdet2-1* mutant has obviously different phenotypes from wild-type in the dark [5]. Hence, we compared the differences of *Atdet2-1* and transgenic lines in the dark. In line with previous reports, different from Col-0, the *Atdet2-1* mutant has a shorter hypocotyl (0.86 cm VS 1.84 cm) and primary roots and open cotyledons (Figure 10A). Overexpression of *GmDET2a* or *GmDET2b* in *Atdet2-1* rescued the hypocotyl growth and primary root growth in the dark. The cotyledons in transgenic lines are less open in contrast to *Atdet2-1*. Data in Figure 10B verified that ectopic expression of *GmDET2a* or *GmDET2b* increases the hypocotyl growth in darkness. Compared to *Atdet2-1*, the hypocotyl length in the *GmET2aOX-20*, *GmDET2aOX-23*, *GmDET2bOX-16* and *GmDET2b-23* lines was increased by 110%, 87%, 146% and 121%, respectively (p<0.05). Taken together, ectopic expression of *GmDET2a* and *GmDET2b* rescued the growth and development defects of *Atdet2-1* in both the dark and light conditions.

### 2.7. Over-Expression of *GmDET2a* or *GmDET2b* Rescued the Responses of BR Biosynthesis-Related Genes to Exogenous BR

As reported, BR biosynthesis-related genes are feedback regulated by BR [47]. Therefore, we sought to determine whether the expression of *GmDET2a* or *GmDET2b* in the *Atdet2-1* mutant background could rescue the responses of BR biosynthesis-related genes *CPD*, *DWF4*, *BR6ox-1* and *AtBR6ox-2*.

**Figure 11:**
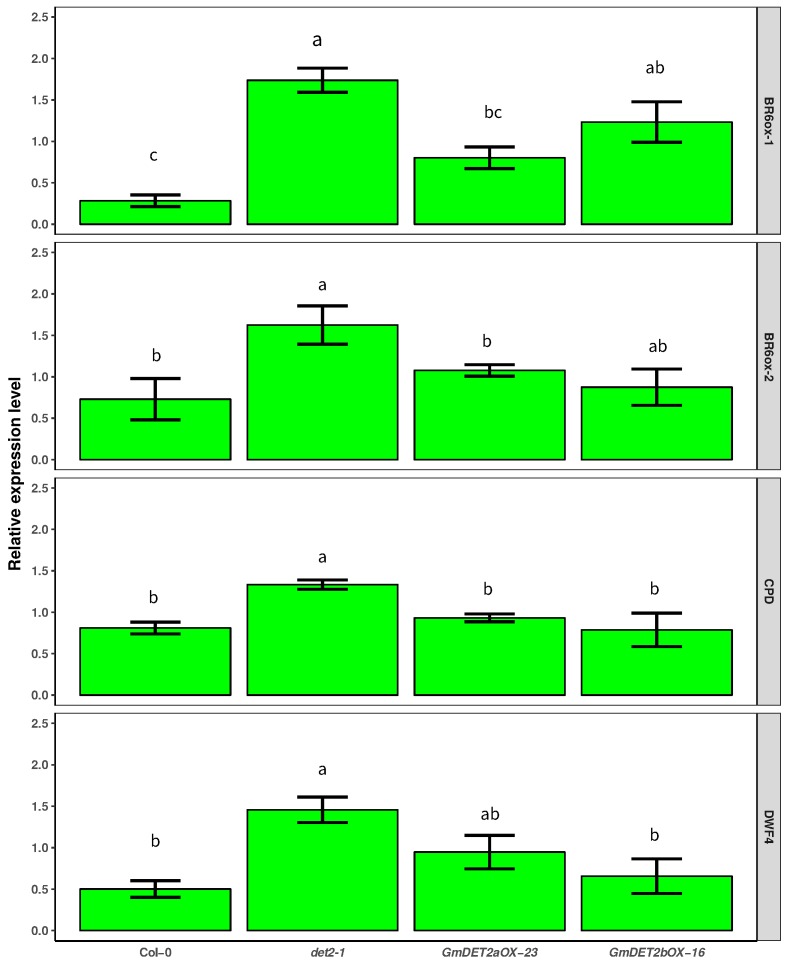
Effect of ectopic over-expression of *GmDET2a* and *GmDET2b* on the transcripts of BR biosynthesis-related genes in the *Atdet2-1* mutant. *Arabidopsis* seedlings were grown as described in the Materials and Methods. Quantitative RT-PCR was used to quantify the relative expression levels of *BR6ox-1* (**A**), *BR6ox-2* (**B**), *CPD* (**C**) and *DWF4* (**D**) in *det2-1* and transgenic lines. Results are the means ± SD from four independent experiments with three technical repeats. One-way ANOVA was used to compare the difference among lines, with different letters indicating significant differences at the 0.05 level.

As demonstrated in Figure 11, compared to WT, the abundances of *BR6ox-1*, *BR6ox-2*, *CPD* and *DWF4* were relative higher in the *det2-1* mutant. When overexpressing *GmDET2a* in *Atdet2-1*, *BR6ox-1*, *BR6ox-2*, *CPD* and *DWF4* transcripts were significantly reduced (p<0.05); with similar results observed when overexpresing *GmDET2b* in *Atdet2-1*. These results verified that soybean *DET2a* and *DET2b* complement the function of *AtDET2* at the molecular level.

## 3. Discussion

Brassinosteroids are classic phytohormones, playing crucial roles in plant growth, development, abiotic and biotic stress responses [12]. Although the BR biosynthesis pathway in *Arabidopsis* has been extensively studied, the understanding of BR biosynthesis in soybean is poor. In this study, we identified eight soybean steroid reductase genes (*GmDET2a* to *GmDET2h*) and functionally characterized *GmDET2a* and *GmDET2b* at the physiological and molecular levels.

As early as 1990, BR was reported to stimulate adventitious rooting in soybean hypocotyl cuttings [37], and BR regulates nodulation and promotes drought tolerance [39,47]. The soybean genome encodes six BR receptors, and GmBRI1a and GmBRI1b have been reported [31,32]. Genes involved in soybean BR biosynthesis, unfortunately, are poorly understood. The BR biosynthetic process can be divided into two stages. The first stage sees the conversion of cycloartenol to campesterol, catalyzed by SMT1, FACKEL, DWF7/TE1, DWF5 and DWF1/DIM1 [47]. The second stage sees the conversion of campesterol to brassinosteroid, catalyzed by SAX1, DET2, DWF4, CPD, BR6ox-1 and BR6ox-2, respectively [47]. *Arabidopsis* DET2 has 80% identity with animal steroid 5α-dehydrogenase, including a highly conserved glutamate required for enzyme activity [35]. In *Atdet2-1* and *Atdet2-6* mutants, the conserved glutamate has been mutated into lysine, and *Atdet2-1* has a 90% reduction in BR as compared to WT [35].

BR biosynthesis has been documented, thus far, in 61 plant species, including 53 angiosperm, 6 gymnosperms, 1 pteridophyte (*Equisetum arvense*) and one bryophyte (*Marchantia polymorpha*) [48]. Single-celled green freshwater algae (*Chlorella vulgaris* and *Hydrodictyon reticulatum*) and the marine brown alga *Cystoseira myrica* biosynthesize BRs, as well [49], indicating that BRs are highly conserved phytohormone. Based on studies in *Arabidopsis* and rice, *DET2*, encoding steroid reductase, a crucial enzyme in BR biosynthesis, catalyzes the rate-limiting step reaction [47]. Homologues of *DET2* in tomato and cotton have been functionally identified, as well [50,51]

In this study, we found that the soybean genome encodes eight steroid reductase (Table 1). This indicates that brassinosteroid biosynthesis in soybean is more complex than in other species. *GmDET2a* and *GmDET2b* are intron-less genes, and consistent with AtDET2, GmDET2a and GmDET2b do not have the PF02544 steroid reductase signature (Table 1). Transgenic experiments verified that *GmDET2a* and *GmDET2b* function as steroid reductases in *Arabidopsis*, but more studies are needed to reveal the functions of the other six soybean *DET* genes. We also used PF02544 to search against the Phytozome database and retrieved 144 proteins containing steroid reductase/dehydrogenase domains from 19 species (Additional File 1). Evaluated by Prottest-2.4, JTT + G + F was selected as the best model to rebuild the evolutionary tree, utilizing MrBayes 3.2 software. As shown in Figure 3, steroid reductases were grouped into five subgroups. Except for Subgroup V, the other four subgroups contain steroid reductases from both *Physcomitrella patens* and *Selaginella moellendorffii*, implying that at least four ancestral steroid reductase genes exist in Plantae. Of note, fungus *Ustilago maydis* steroid 5α-reductase complements *Arabidopsis* DET2 function [52], and overexpressing a human steroid 5α-reductase rescues the growth and development of *Atdet2-1* [35]. This evidence further verifies the existence of a common ancestor for steroid reductase between fungal, plant and mammalian proteins.

We found that in most branches, the Ka/Ks of steroid reductases was less than 1.0 (Figure 4), indicating that steroid reductases have been subjected to purifying selection. Ka/Ks values are also consistently less than one in BR receptor genes [32]. Similarly, as high as 76% analyzed jasmonate ZIM-domain (JAZ) genes from 13 monocot and dicot species have also been subject to purifying selection [53], and the Ka/Ks of the squamosa promoter binding protein (SBP)-box genes in plants were less than 0.5 [54]. Our results support the notion that the Ka/Ks of crucial genes in many plant species are less than 1.0 [55].

RNA-Seq data indicate that transcripts of eight soybean *DET* genes can be detected in roots, leaves, flowers, seeds and nodules (Figure 1B), implying that BR regulates the development and growth of these organs. Notably, the transcript abundances of *GmDET2a* and *GmDET2b* are higher in nodules and roots, indicating their important role in nodulation and rooting. The interaction between BR and auxin has been shown to stimulate lateral rooting [56], and BR signaling has been proven to boost root hair development [57].

Similarly, in tomato, the transcripts of *LeDET2* can be detected in leaves, stems, roots, seeds and callus, with the highest abundance observed in leaves [50]. In this study, qRT-PCR results revealed that *GmDET2a* and *GmDET2b* have similar expression patterns (Figure 5) with higher expression in primary root tips, apical buds and lateral roots, implying that BR biosynthesis is active in these tissues. Differing from auxin, BR does not experience polar transport; thus, it is understandable that *DET2* shows ubiquitous expression in primary roots, lateral roots, seeds and nodules. The future challenge will be to reveal the differing roles of soybean *DET2s* in different organs.

We found that application of EBL for 6 h significantly decreased the transcripts of *GmDET2a* and *GmDET2b* in apical buds, leaves, PR tips and lateral roots (Figure 6A,B). *DET2*, *CPD*, *DWF4*, *BR6ox-1* and *BR6ox-2* are key enzymes for BR biosynthesis [47] and are negatively regulated by exogenous BR or BR signal [47,58]. In line with this, we found that the transcripts of *CPD*, *DWF4*, *BR6ox-1* and *BR6ox-2* were downregulated in WT in contrast to *Atdet2-1*, but overexpressing *GmDET2a* or *GmDET2b* negatively regulated the levels of these four genes in *Atdet2-1* (Figure 11). This is possibly due to the fact that ectopic expression of *GmDET2a* or *GmDET2b* in *Atdet2-1* increases endogenous BR amounts. Molecular evidence also supports the idea that *GmDET2a* or *GmDET2b* encodes steroid reductase. Transcripts of *DWF4* in WT are low, but those of *DWF1*, *CPD* and *BRI1* are higher, indicating that transcription of *DWF4* is downregulated by BR [47]. The fact that *Arabidopsis CPD*, *DWF4* promoters contain binding elements of BZR1 or BES1 prompted us to conceive of the idea that the same *cis*-elements also exist in the promoter region of *GmDET2a* and *GmDET2b*. The homologue of BES1 or BZR1 can be blasted in soybean proteome.

Phosphate starvation induces the expression of *GmDET2b*, and S starvation downregulates the transcripts of *GmDET2a* and *GmDET2b* (Figure 7). This hints at potential crosstalk between nutrients and BR biosynthesis. Whether it is phosphate- or sulfate-related transcription factors binding the promoters of *GmDET2a* or *GmDET2b* would be worthy of future study.

*Atdet2-1* mutants exhibit a dwarf phenotype, and in darkness, the hypocotyl length of *det2-1* is shorter than WT [5]. By ectopically expressing *GmDET2a* or *GmDET2b* in *Atdet2-1*, we show that GmDET2a and GmDET2b function as steroid reductase, as their overexpression rescued the defects of the mutant (curled leaf, reduced plant height in light and reduced hypocotyl length in dark) (Figure 8, Figure 9 and Figure 10). In conclusion, we functionally identified GmDET2a and GmDET2b as steroid reductases involved in BR biosynthesis.

## 4. Materials and Methods

### 4.1. Soybean and *Arabidopsis* Growth

*Glycine max* genotype YC03-3 was used in this study. Soybean seeds were sterilized with 10% NaClO and germinated in sand until the cotyledon was open. The seedlings were then transferred to Hoagland solutions (pH = 5.9) for three days, until the first true leaf developed. The seedlings were then cultured in 10 μM EBL solution for 0 h and 6h, and the leaves were sprayed with 10 μM EBL (+EBL) or 0 μM EBL (-EBL). The apical buds, true leaves, hypocotyls, primary root tips and lateral roots were then removed and stored in liquid nitrogen. To study nutrient stress, germinating soybean seedlings were first cultured in complete Hoagland solution (pH = 5.9) for seven days, as described [59], and the seedlings with the first fully-developed trifoliate leaves were transferred into phosphate (Pi)-sufficient (Control, 250 μM ${\mathrm{KH}}_{2}{\mathrm{PO}}_{4}$) or phosphate deficient (-P) (0 μM ${\mathrm{KH}}_{2}{\mathrm{PO}}_{4}$) nutrient solution, for 14 days. For nitrogen starvation (-N), ${\mathrm{NH}}_{4}{\mathrm{NO}}_{3}$ was omitted, and KCl was substituted for ${\mathrm{KNO}}_{3}$. For potassium depletion (-K), ${\mathrm{KNO}}_{3}$ was omitted. For sulfate deficiency(-S), all ${\mathrm{SO}}_{4}^{2-}$ was substituted with ${\mathrm{Cl}}^{-1}$. All soybean solutions were aerated 15 min every 3 h and replaced with fresh solution every 48 h. Soybean plants were grown under greenhouse conditions with 16-h light cycles.

*Arabidopsis* seeds were sterilized with 75% ethanol for 2 min followed by 100% ethanol for 2 min. Seeds were dried in a hood and sown on half strength MS media containing 1% sucrose and 0.8% agar (pH 5.8). Square Petri dishes were maintained in a growth chamber with a 16-h light (100 μE m${}^{-2}$ s${}^{-1}$)/8-h dark cycle and with a temperature cycle of 23 ${}^{\circ }$C light/21 ${}^{\circ }$C dark. For the darkness treatment, the square Petri dishes were wrapped with aluminum foil.

### 4.2. Extraction of Genomic DNA, RNA and Reserve Transcription of mRNA

*Arabidopsis* and soybean RNA were extracted with the TRIzol method. RNA were reverse transcribed through MLV-transcriptase.

### 4.3. Determination of Soybean *GmDET2* Family Structure

Based on the full cDNA and genomic DNA sequences of *GmDET2a to DET2h* (www.phytozome.org), we exploited CSDS2.0 to plot the gene structure of GmDET2s.

### 4.4. Alignment of DET2s

T-coffee (http://www.tcoffee.org/Projects/tcoffee/) was employed to align the sequence of DET2 from *Glycine max*, *Arabidopsis*, *Solanum lycopersicum*, *Oryza sativa*, *Pisum sativum*, *Hordeum vulgare* and *Medicago truncatula*.

### 4.5. Phylogenetic Analysis

Alignment of steroid reductases from *Glycine max*, *Arabidopsis* and *Oryza sativa* was done with ClustalW2.1. The evolutionary history was inferred by using the maximum likelihood method based on the Jones et al. w/freq.model [44]. The tree with the highest log likelihood (−2073.6839) is shown. The percentage of trees in which the associated taxa clustered together is shown next to the branches. Initial trees for the heuristic search were obtained automatically by applying neighbor-join and BioNJalgorithms to a matrix of pairwise distances estimated using a JTT model and then selecting the topology with the superior log likelihood value. A discrete gamma distribution was used to model evolutionary rate differences among sites (5 categories (+G, parameter = 5.1876)). The tree is drawn to scale, with branch lengths measured in the number of substitutions per site. The analysis involved 20 amino acid sequences. All positions containing gaps and missing data were eliminated. There was a total of 74 positions in the final dataset. The bootstrap values were set to 1000. Evolutionary analyses were conducted in MEGA6 [42].

A total of 105 steroid reductases from *Physcomitrella patens*, *Selaginella moellendorffii*, *Cucumis sativus*, *Populus trichocarpa*, *Eucalyptus grandis*, *Citrus clementina*, *Solanum lycopersicum*, *Glycine max*, *Arabidopsis thaliana*, *Brachypodium distachyon*, *Oryza sativa*, *Amborella trichopoda*, *Gossypium raimondii*, *Aquilegia coerulea* and *Sorghum bicolor* were selected to reconstruct the Bayesian phylogenies with MrBayes v3.2 [43]. In order to guarantee that the average standard deviation of split frequencies is below 0.05, we set the runs of generation to be 100,000. We employed a fixed Jones model (JTT) of amino acid substitution to reconstruct the phylogenetic tree (other parameters: nchain = 4, samplefreq = 100, samplefreq = 100, printfreq = 100 diagnfreq = 1000).

### 4.6. Analysis of Expression Patterns of Soybean *DET2* Family at the Seedling Stage

Based on the cDNA sequence and genomics sequences of *GmDET2a* and *GmDET2b* (www.phytozome.org), we designed specific primer pairs with PerlPrimer to detect their expression levels. Seven-day-old soybean cultivar YC03-3 seedlings were transferred to half strength Hoagland nutrient solutions to grow a further 7 days treated with different nutrient solutions. The roots, stems and leaves were sampled to extract total RNA. Quantitative real-time PCR was used to test the expression levels of *GmDET2a* and *GmDET2b*, and soybean *EF1a* was used to normalize the data. Forty qRT-PCR cycles were run (95 ${}^{\circ }$C 1 min, 95 ${}^{\circ }$C 15 s, 60 ${}^{\circ }$C 15 s, 72 ${}^{\circ }$C 30 s, 72 ${}^{\circ }$C 45 s), and Rotorgen software was used to calculate the PCR results. Data were from three independent biological experiments.

### 4.7. Overexpression of *GmDET2a and GmDET2b* in *Arabidopsis* *det2*

As the coding region sequences of *GmDET2a* and *GmDET2b* are nearly 100% identical, we did two rounds of PCR to clone the open reading frame of *GmDET2a* and *GmDET2b*. First, we designed PCR primers in the 5 UTR and 3 UTR of *GmDET2a* and *GmDET2b* and then used the common forward and reverse primers GmDET2a/b.oxF and GmDET2a/b.oxR that contain the KpnI and SalI restriction sites, respectively, to amplify the coding fragment. The coding fragments of *GmDET2a* and *GmDET2b* and pCHF3 plasmid were digested with KpnI and SalI, respectively, and the digested pCHF3, *GmDET2a* and *GmDET2b* coding fragments were ligated using DNA ligase. The *Atdet2-1* mutant was transformed with pCHF3-*GmDET2a* or pCHF3-*GmDET2b* using the floral dip method and the GV3101 agrobacterium strain [60]. Transformed lines were selected in media containing 50 μg/mL kanamycin. The single *copy* T-DNA transformed lines were determined based the 3:1 segregation ratio. The homozygous F3 were used in further experiments.

### 4.8. Phenotypic Analysis of Transgenic Arabidopsis

Seeds of wild-type Col-0, *Atdet2-1* and overexpression lines in mutant background, as indicated, were stratified in 4 ${}^{\circ }$C for 2 days to break dormancy and then sown into soils, and after being cultured as indicated, seedling height was measured with a ruler.

### 4.9. Determination of the Expression Levels of BR Biosynthesis-Related Genes in Col-0, *det2-1* and Transgenic Lines

To test the expression levels of BR biosynthesis-related genes *CPD*, *DWF4*, *BR6ox-1* and *BR6ox-2*, 3-week-old *Arabidopsis* seedlings of Col-0, *Atdet2-1* and overexpression lines in mutant background were transplanted to the soil for one month under standard growth conditions, and then, the whole plants were used to extract total RNA with the TRIzol method. cDNAs were obtained with reverse transcriptase-mediated reactions. Transcript abundance of *CPD*, *DWF4*, *BR6ox-1* and *BR6ox-2* was determined using qRT-PCR, and *AtEF1a* was used as a reference gene to normalize the qRT-PCR results. Specific primer pairs are listed in Supplementary Table S2.

### 4.10. Data Analysis

All data were analyzed with Excel2003. Student’s *t*-test and one-way ANOVA were employed to compare the differences. R 3.0.1 package [61], ggplot2 and gplots 2.12.1 [62] were used to draw figures and the heat map, respectively.

## 5. Conclusions

The soybean genome has eight genes that encode steroid reductase (*GmDET2a* to *GmDET2h*), and *GmDET2a* and *GmDET2b* were negatively regulated by exogenous BR. BR reductases in plants were subjected to purifying selection during evolution. Ectopic overexpression of *GmDET2a* and *GmDET2b* in *Atdet2-1* rescues the BR biosynthesis deficiency-related growth and development defects of the *det2-1* under dark and light conditions.

## Figures and Tables

**Table 1:** General information about the steroid reductases in soybean based on bioinformatics analysis.

Name	Locus	Alias	EST	Length (AA)	Putative Localization	Int/Ext
*GmDET2a*	Glyma.07G144400	Glyma07g17410	yes	263	Plas	0/1
*GmDET2b*	Glyma.11G010000	Glyma11g01210	yes	263	Plas	0/1
*GmDET2c*	Glyma.02G081200	Glyma02g08970	yes	266	Cyto	1/2
*GmDET2d*	Glyma.02G081100	Glyma02g08950	yes	266	Plas	1/2
*GmDET2e*	Glyma.11G110300	Glyma11g11780	yes	342	Nucl	5/6
*GmDET2f*	Glyma.14G043500	Glyma14g04780	yes	309	Plas	3/4
*GmDET2g*	Glyma.02g273300	Glyma02g44090	yes	309	?	4/5
*GmDET2h*	Glyma.19g261600	Glyma19g45120	yes	317	?	2/3

Putative cell localization of soybean steroid reductases was predicted with PSORT. Based on the released genome sequences and cDNA sequences of soybean, the number of introns and exons were determined through GSDS as described in the Materials and Methods. EST, expressed sequence tag; Plas, plasma membrane; Nucl, nucleus; Cyto, cytoplasm; AA, amino acid; Int, intron; Ext, extron.

## Supplementary Materials

The following are available online at http://www.mdpi.com/1422-0067/19/3/726/s1.

## Abbreviations

AA	amino acid
BAK1	BRI1-associated receptor kinase 1
BIN2	brassinosteroid insensitive 2
BKI1	BRI1 kinase inhibitor 1
BR	brassinosteroid
BRI1	brassinosteroid insensitive 1
Brz	brassinazole
BZR1	brassinazole-resistant 1
d	day
h	hour
ID	island domain
K	potassium
N	nitrogen
ORF	open reading frame
P	phosphorus
PR	primary root
qRT-PCR	quantitative real-time PCR
S	sulfate
