# Supplementary material for: Functional Characterization of the Steroid Reductase Genes GmDET2a and GmDET2b from Glycine max
Source: Int J Mol Sci. 2018 Mar 3;19(3):726. doi: 10.3390/ijms19030726 (PMC5877587; doi:10.3390/ijms19030726)
Supplement: Supplementary file 1 [file ijms-19-00726-s001.zip › Table_S2.pdf]

Table S1 List of primers in this study

GmDET2a.nF	*TCACATATTTTCGTCCATTACCA*	Nest-PCR to amplify *Gm DET2a*
GmDET2a.nR	TGTCCTTTGAAGTAGCAGTGTTA	Nest-PCR to amplify *Gm DET2a*
GmDET2b .nF	TCCATCTTCTCTAATCTCCGTC	Nest-PCR to amplify *Gm DET2b*
GmDET2b.nR	CCTTTGAAGTAGCTAGTCAGCT	Nest-PCR to amplify *Gm DET2b*
GmDET2a/2b.oxF	AaggtaccATGATCCCAGAACACTACTCC	Over-expression
GmDET2a/2b.oxR	AagtcgacTCAATACAAGTAAGGAATAACAGC	Over-expression
AtCPD.qF	TTGCTCAACTCAAGGAAGAG	qRT-PCR
AtCPD.qR	TGATGTTAGCCACTCGTAGC	qRT-PCR
AtDWF4.qF	CATAAAGCTCTTCAGTCACGA	qRT-PCR
AtDWF4.qR	CGTCTGTTCTTTGTTTCCTAA	qRT-PCR
AtBR6ox1.qF	TCCCGTATCGGAGTCTTTGGT	qRT-PCR
AtBR6ox1.qR	TGGCCAATCTTTGGCGAA	qRT-PCR
AtBR6ox2.qF	CAATAGTCTCAATGGACGCAGAGT	qRT-PCR
AtBR6ox2.qR	AACCGCAGCTATGTTGCATG	qRT-PCR
GmDET2a.qF	ACCGTAGGTGGTATTTGGAG	qRT-PCR
GmDET2a.qR	TCCTTGACCTCACATTAAGCA	qRT-PCR
GmDET2b.qF	GGTGGTATTTGGAGAAGTTTGG	qRT-PCR
GmDET2b.qR	CCCACATTCCCAAATACAATCC	qRT-PCR
AtEF1-α 4.qF	CTGGAGGTTTTGAGGCTGGTAT	qRT-PCR
AtEF1-α 4.qR	CCAAGGGTGAAAGCAAGAAGA	qRT-PCR
GmEF-1 α .qF	CTGGAGGTTTTGAGGCTGGTAT	qRT-PCR
GmEF-1 α .qR	CCAAGGGTGAAAGCAAGAAGA	qRT-PCR
GmDET2a/b.qF	AATGTATGGGCTGATAGGGT	qRT-PCR to identify over-expression lines of GmDET2aOX or GmDET2bOX
GmDET2a/b.qR	AGGTGGTATTTGGAGAAGTTTG	qRT-PCR to identify over-expression lines of GmDET2aOX or GmDET2bOX
