# Supplementary figures and images for: Functional Characterization of the Steroid Reductase Genes GmDET2a and GmDET2b from Glycine max
Source: Int J Mol Sci. 2018 Mar 3;19(3):726. doi: 10.3390/ijms19030726 (PMC5877587; doi:10.3390/ijms19030726)

**A**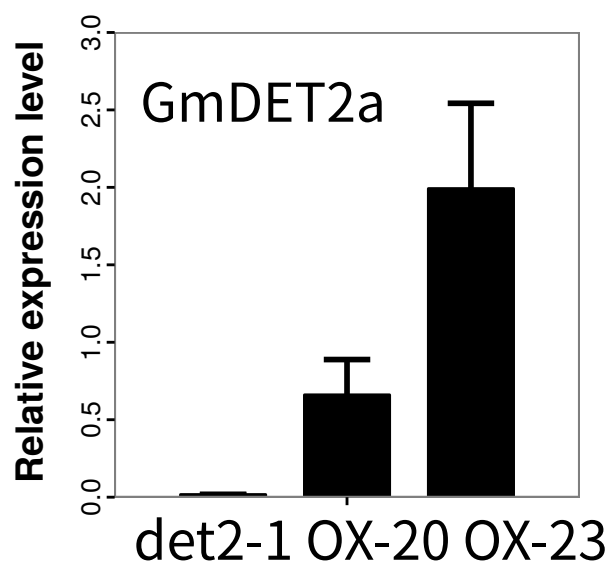**B**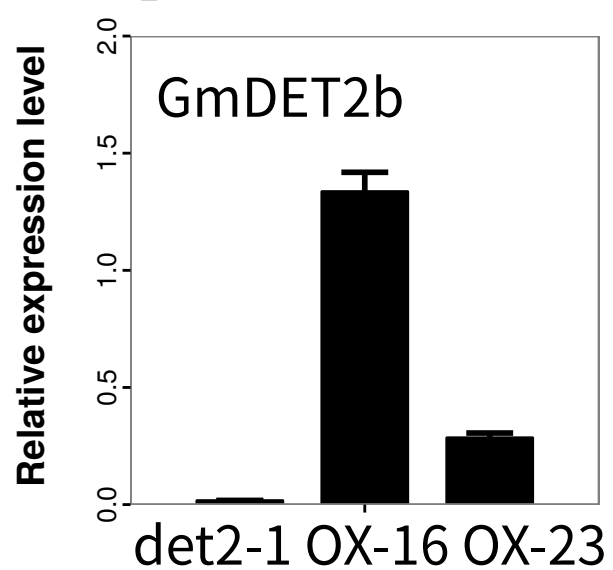

Supplement: Supplementary file 1 [file ijms-19-00726-s001.zip › Fig_S1.pdf]
